# Comparing two implementation strategies for implementing and sustaining a case management practice serving homeless-experienced veterans: a protocol for a type 3 hybrid cluster-randomized trial

**DOI:** 10.1186/s13012-022-01236-1

**Published:** 2022-10-03

**Authors:** Sonya Gabrielian, Erin P. Finley, David A. Ganz, Jenny M. Barnard, Nicholas J. Jackson, Ann Elizabeth Montgomery, Richard E. Nelson, Kristina M. Cordasco

**Affiliations:** 1 HSR&D Center for the Study of Healthcare Innovation, Implementation and Policy (CSHIIP), VA Greater Los Angeles Healthcare System, North Hills, CA USA; 2 Desert Pacific Mental Illness Research, Education, and Clinical Center (MIRECC), VA Greater Los Angeles, Los Angeles, CA USA; 3 Department of Psychiatry, VA Greater Los Angeles Healthcare System, Los Angeles, CA USA; 4 Department of Psychiatry and Biobehavioral Sciences, David Geffen School of Medicine, University of California at Los Angeles, Los Angeles, CA USA; 5 Division of Hospital Medicine, Department of Medicine and Department of Psychiatry, University of Texas Health San Antonio, San Antonio, TX USA; 6 Department of Medicine, VA Greater Los Angeles Healthcare System, Los Angeles, CA USA; 7 Greater Los Angeles Geriatric Research, Education, and Clinical Center (GRECC), VA Greater Los Angeles, Los Angeles, CA USA; 8 Department of Medicine, David Geffen School of Medicine, University of California at Los Angeles, Los Angeles, CA USA; 9 Department of Medicine Statistics Core, David Geffen School of Medicine, University of California at Los Angeles, Los Angeles, CA USA; 10 Birmingham Veterans Affairs Health Care System, Birmingham, AL USA; 11 School of Public Health, University of Alabama at Birmingham, Birmingham, AL USA; 12 Veterans Affairs Salt Lake City Health Care System, Salt Lake City, UT USA; 13 Department of Internal Medicine, University of Utah School of Medicine, Salt Lake City, UT USA

**Keywords:** Homeless veterans, Case management, Replicating effective programs, Facilitation, Implementation science

## Abstract

**Background:**

The Veterans Health Administration (VA) Grant and Per Diem case management “aftercare” program provides 6 months of case management for homeless-experienced veterans (HEVs) undergoing housing transitions. To standardize and improve aftercare services, we will implement critical time intervention (CTI), an evidence-based, structured, and time-limited case management practice. We will use two strategies to support the implementation and sustainment of CTI at 32 aftercare sites, conduct a mixed-methods evaluation of this implementation initiative, and generate a business case analysis and implementation playbook to support the continued spread and sustainment of CTI in aftercare.

**Methods:**

We will use the Replicating Effective Programs (REP) implementation strategy to support CTI implementation at 32 sites selected by our partners. Half (*n*=16) of these sites will also receive 9 months of external facilitation (EF, enhanced REP). We will conduct a type 3 hybrid cluster-randomized trial to compare the impacts of REP versus enhanced REP. We will cluster potential sites into three implementation cohorts staggered in 9-month intervals. Within each cohort, we will use permuted block randomization to balance key site characteristics among sites receiving REP versus enhanced REP; sites will not be blinded to their assigned strategy. We will use mixed methods to assess the impacts of the implementation strategies. As fidelity to CTI influences its effectiveness, fidelity to CTI is our primary outcome, followed by sustainment, quality metrics, and costs. We hypothesize that enhanced REP will have higher costs than REP alone, but will result in stronger CTI fidelity, sustainment, and quality metrics, leading to a business case for enhanced REP. This work will lead to products that will support our partners in spreading and sustaining CTI in aftercare.

**Discussion:**

Implementing CTI within aftercare holds the potential to enhance HEVs’ housing and health outcomes. Understanding effective strategies to support CTI implementation could assist with a larger CTI roll-out within aftercare and support the implementation of other case management practices within and outside VA.

**Trial registration:**

This project was registered with ClinicalTrials.gov as “Implementing and sustaining Critical Time Intervention in case management programs for homeless-experienced Veterans.” Trial registration NCT05312229, registered April 4, 2022.

Contributions to the literature

- This protocol presents a pragmatic example of implementing a complex case management practice in diverse community-based organizational settings.
- This trial integrates a cluster-randomized design and mixed-methods evaluation to compare the impacts of two implementation strategies on the implementation and sustainment of an evidence-based case management practice.
- This protocol describes the development of products (i.e., business case analysis and implementation playbook) that can be used by policy partners to support the continued spread and sustainment of a complex case management practice in diverse community-based organizations.

## Background

Compared to housed veterans, homeless-experienced veterans (HEVs) have profound health disparities, including high rates of medical illness, psychiatric problems, and substance use disorders (SUDs) [1]. With a substantial investment of VA resources, veteran homelessness decreased by 50% (from 73,367 to 37,252) from 2009 to 2020 [2]. Veterans who remain homeless despite these services are extraordinarily vulnerable; 40% are unsheltered, living outdoors or in other places not intended for human habitation [2]. To further the VA’s goal of ending veteran homelessness [3], there is a pressing need to identify strategies that support the scale-up, spread, and sustainment of evidence-based practices (EBPs) across the range of programs that serve HEVs.

The Grant and Per Diem (GPD) program is a cornerstone of the VA’s plan to end veteran homelessness. Operated by VA’s community partners, this program provides transitional housing (for ≤24 months) and supportive services for HEVs. Many HEVs transitions from GPD sites into independent housing; the initial phase of this transition can be challenging and associated with adverse outcomes, including returns to homelessness and hospitalizations [4]. Until recently, GPD case management ceased during the transition from GPDs, leaving HEVs without structured case management unless they entered VA’s permanent supportive housing program. In March 2019, under direction from Congress, VA awarded $30 million to 128 GPD sites to provide 6 months of case management (in the GPD case management “aftercare” program) for HEVs transitioning to independent living and not otherwise receiving case management. However, no specific case management paradigm was required in aftercare, resulting in site-level practice variation.

GPD National Program Office policy leaders identified critical time intervention (CTI) as an evidence-based, structured, and time-limited case management model that—if implemented nationally—would standardize and improve aftercare case management. There are five randomized controlled trials (RCTs) [5–8] and a systematic review [9] demonstrating that CTI effectively improves housing and decreases hospitalizations among homeless-experienced adults. Moreover, CTI was successfully implemented in 8 VA facilities for HEVs with serious mental illness, suggesting it is feasible and appropriate for VA scale-up and spread. CTI is also well-aligned with aftercare, which was designed to improve housing stability among HEVs undergoing housing transitions. However, implementing EBPs in community-based organizations serving HEVs brings challenges [10, 11].

This paper describes the protocol for the Housing Transitions Quality Enhancement Research Initiative (QUERI), which will implement CTI across 32 aftercare sites nationally and compare the impacts of two implementation strategies on effectiveness and implementation outcomes. To implement and sustain CTI, we will use the Replicating Effective Programs (REP) implementation bundle to enable sites to achieve fidelity to CTI’s core components, while accommodating adaptations to fit the diversity of aftercare contexts. REP includes EBP packaging, staff training and technical assistance, process evaluation and feedback, and ongoing maintenance support [12]. Half of these sites (*n*=16) will receive an “enhanced REP” bundle in which REP is augmented by 9 months of EF, a process of providing tailored support for providers and leaders to adopt and incorporate EBPs into routine care. We will provide our partners with evidence regarding the comparative impacts and costs of REP vs. enhanced REP for CTI implementation and sustainment across diverse aftercare contexts, including sites with quality gaps.

Partnered with national and regional leaders in VA homeless services, this project’s Specific Aims are to [1] use REP and enhanced REP to support the implementation and sustainment of CTI in 32 aftercare sites [2]; compare, in a type 3 hybrid implementation-effectiveness trial [13], the impacts of REP vs. enhanced REP on CTI fidelity and sustainment, quality metrics (focused on housing stability and hospitalizations), and costs and return-on-investment; and [3] generate two key products for program partners—a business case analysis and an implementation playbook—to support continued spread and sustainment of CTI in the aftercare program.

## Methods

### Overview

We hypothesized that enhanced REP will have higher implementation costs than REP, but enable increased tailoring to local contexts that results in stronger CTI implementation and effectiveness, supporting a business case for enhanced REP. To test this hypothesis, we will use a type 3 hybrid implementation-effectiveness trial [13]; this design tests the impacts of implementation strategies while gathering data regarding an EBP’s impacts on clinical outcomes. Specifically, we will compare the impacts of REP versus enhanced REP on CTI implementation and sustainment, while observing CTI’s effectiveness on housing and health outcomes. In a cluster randomized design, we will assign 32 aftercare sites to REP (*n*=16) versus enhanced REP (*n*=16), clustering sites in three implementation cohorts staggered in 9-month intervals.

### Conceptual framework

**Fig. 1:**
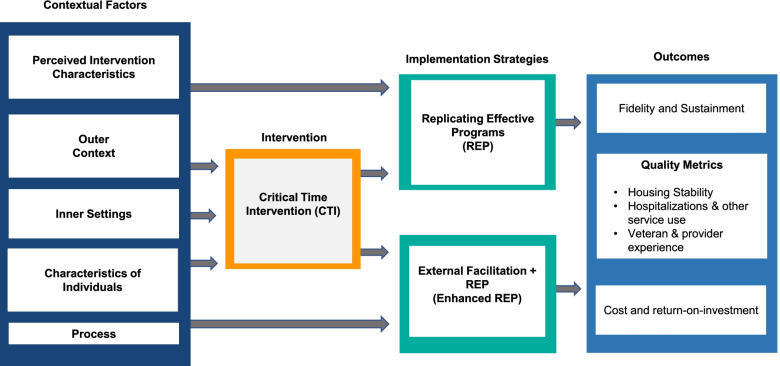
Conceptual framework [15, 16]

Housing Transitions QUERI is built on the conceptual framework illustrated in Fig. 1, which draws upon recommendations by Smith et al. [14] for specifying the logic in implementation planning and evaluation. We used the Consolidated Framework for Implementation Research (CFIR) [15], which draws upon theory and constructs from implementation science to describe contextual factors across five domains (perceived intervention characteristics, outer context, inner setting, characteristics of individuals, and implementation processes) associated with CTI implementation outcomes.

### Critical time intervention (CTI)

**Fig. 2:**
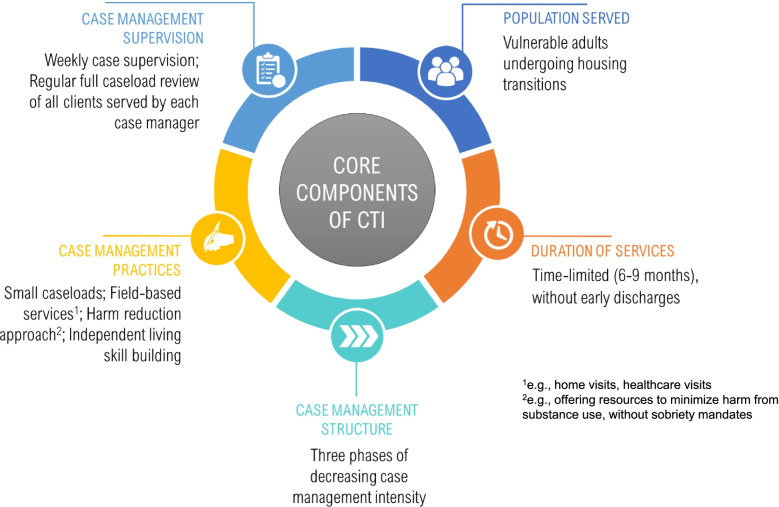
Core components of CTI

In CTI, clients (e.g., HEVs) are assigned to case managers (“CTI specialists”) who link them to services and resources aligned with their housing and other goals. Core components of CTI (Fig. 2) include the CTI specialist who delivers field-based services (e.g., home visits) that help clients mobilize resources and support. The background and training of CTI specialists range from peer providers with lived expertise in homelessness to master’s level clinicians (e.g., social workers). CTI is delivered over 6 to 9 months in three time-limited phases of decreasing intensity: [1] transition to the community (development of a care plan with salient goals), [2] try out (clients test problem-solving skills using plans established with their CTI specialist), and [2] transfer of care (care transition from CTI specialist to a longitudinal care team). Services focus on housing stability, personal goals, and building a support network. The Template for Intervention Description and Replication (TIDiER) checklist is available as supplemental material.

### CTI implementation pilot

Housing Transitions QUERI builds on a 1-year implementation pilot in which we developed resources and processes for CTI training and technical assistance (TA) and external facilitation that were piloted and refined at four aftercare sites. We also identified outcome measures for future evaluation. To this end, we formed a virtual stakeholder workgroup comprised of two CTI expert trainers, a faculty consultant from a university-based center with CTI expertise, a VA social worker who served as the “liaison” to the four pilot sites, and a peer provider with professional and lived expertise in GPD programs.

We drew upon this workgroup to tailor a 6-month version of CTI that was previously implemented in Connecticut-based homeless programs [17]. We made practice adaptations to reflect the aftercare context and augmented anonymized cases presented in the training and TA materials to reflect the social circumstances, functioning, and diagnoses of HEVs in aftercare. We made additional adaptations in response to precautions imposed by the coronavirus disease 2019 (COVID-19) pandemic and ensured that all training and TA could be delivered virtually. We also developed an online toolkit [18] with clinical resources, recorded trainings, a CTI manual, and training and TA slide decks.

Next, we pilot-tested this adapted training and TA package (Table 1) with aftercare case managers, supervisors, and our VA social work liaison. First, initial CTI training was delivered in six synchronous videoconferences by national leaders in training and consultation for case managers serving homeless-experienced adults. Next, we initiated a monthly community of practice sessions, i.e., 1-hour discussions to deepen knowledge and expertise in CTI; these sessions included a presentation from the community of practice leader (a social worker on our team) or guest speaker, followed by a moderated discussion among aftercare staff. We also offered individual, on-demand, 30-min case consultations to aftercare staff with a CTI-trained clinician expert on our team.

**Table 1:** CTI training and technical assistance (TA) package tailored in the Housing Transitions QUERI implementation pilot

Training/TA component	When is the component delivered?
Six session initial CTI training delivered via synchronous videoconference (2 h/week for 6 weeks)	Once, at the start of CTI implementation, for all aftercare case managers and supervisors
Online CTI toolkit, including recorded training sessions, the CTI manual, clinical templates and tools, and relevant resources	As needed by any aftercare case manager or supervisor
On-demand case consultation process with a CTI expert via telephone or videoconference (30 min/consultation)	As needed—requested by any aftercare case manager or supervisor, up to once/month per aftercare site
Community of practice sessions delivered via synchronous videoconference (1 h/session)	Monthly for 6 months, starting the month after the 6-session initial CTI training is completed
Listserv to facilitate sharing of clinical practices and anonymized case discussion among aftercare case managers and supervisors across sites	As needed by any aftercare case manager or supervisor
CTI booster sessions delivered via synchronous videoconference (1 h/training)	Every 3 months, beginning 9 months after the start of CTI implementation

Last, we developed and pilot-tested EF materials and processes. EF is a flexible implementation strategy involving deliberate and interactive problem-solving and support for providers to implement EBPs. In our pilot, a facilitator trained in CTI and facilitation provided tailored support that built the sites’ organizational capacity to implement CTI and empowered case managers to enact systems-based change toward CTI implementation [19]. After each session, the facilitator completed a templated form to summarize the call, engage in self-reflection, delineate facilitation strategies used, and highlight barriers faced in employing these strategies. Data from these forms were used to create a CTI facilitation guide.

Qualitative data from this pilot suggested that most aftercare case managers were highly satisfied with CTI and comfortable using this practice with HEVs. Post-training, case managers reported that CTI was acceptable and credible, with strong data for its effectiveness. Though case managers struggled with the time-limited (6 months) aftercare duration, there was consensus that CTI was a compatible and useful model in this context. Funding was obtained to proceed to a 32-site national implementation and evaluation.

### Implementation strategy: Replicating Effective Programs (REP)

Housing Transitions QUERI will use REP at 32 aftercare sites to implement CTI. REP is a bundle of implementation strategies with a strong evidence base and precedent of feasibility within VA [20, 21]. REP uses stakeholder input to facilitate packaging, training, and TA of EBPs; we selected REP because it resembles “usual care” mental health EBP rollouts in VA. Our use of REP is intended to enhance case managers’ CTI skills and clinical competency, thereby strengthening CTI fidelity and sustainment. REP is particularly well-suited for CTI implementation; research shows that adapting and packaging CTI and its trainings and TA for program and population contexts, using stakeholder input, is critical for effective, high-fidelity implementation [22].

**Fig. 3:**
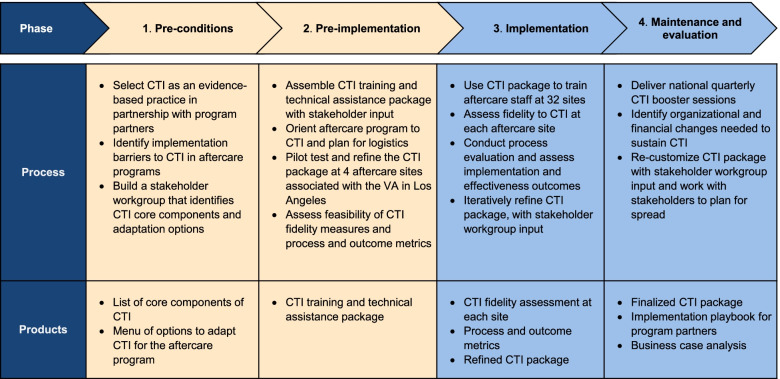
Replicating effective programs (REP) specified for CTI implementation in the aftercare program

Figure 3 depicts REP’s phases and each phase’s products, specified for this project. Phases one and two (“pre-conditions and “pre-implementation”) were completed in our implementation pilot. Phases three and four comprise the focus of this protocol. Phase three (“implementation”) will encompass national delivery of the CTI package to 32 sites, with iterative refinement informed by our implementation evaluation. Phase four (“maintenance and evolution”) will include booster sessions, modeled on prior CTI rollouts. This final phase of REP also includes assessing financial factors and organizational changes needed for CTI sustainment.

Of note, though REP enables stakeholder-informed CTI training and TA, it does not include site-level support beyond on-demand case consultation. Aftercare sites that receive only REP will not work with our team to develop site-level implementation support. As such, REP alone does not provide support specifically tailored to local contexts.

### Implementation strategy: enhanced REP

**Fig. 4:**
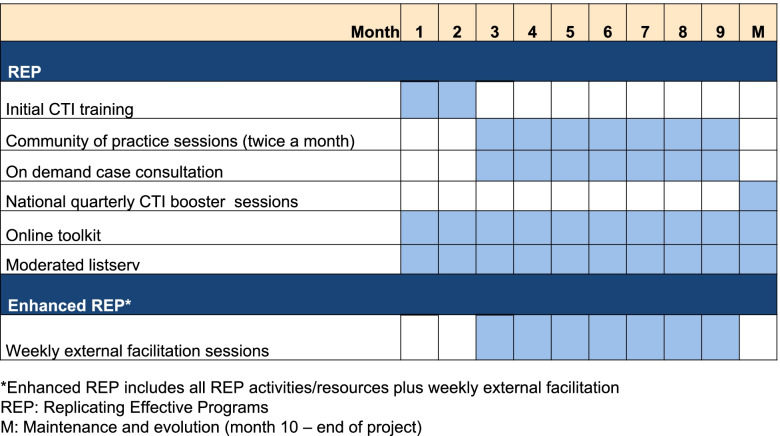
REP vs. enhanced REP, as experienced by sites

In addition to REP, 16 of 32 aftercare sites will receive EF (enhanced REP), which will provide these sites with support tailored to their local contexts. Compared to REP alone, REP enhanced with EF is a higher intensity and higher cost strategy to support CTI implementation. Figure 4 depicts REP versus enhanced REP activities as experienced by sites. We hypothesize that enhancing REP with EF will improve CTI fidelity and sustainment by catalyzing case management activation and organizational changes.

We selected EF to enhance REP because it is a powerful tool, based on organizational theory, that can assist with organizational change by addressing site-specific implementation barriers. In our pilot, we found that tailored support for site-specific challenges was important for CTI implementation. In Housing Transitions QUERI, EF will be delivered by facilitators who are social workers trained in CTI and facilitation. EF sessions (30 min/week videoconferences) will begin within 2 weeks of aftercare staff completing initial CTI training and continue through month 9 of CTI implementation. The content of sessions will vary by sites’ needs and contextual factors; facilitators will employ the guide developed in our pilot, using real-time data and quality improvement techniques to identify steps that sites can follow to implement CTI.

Throughout, facilitators will engage in the implementation and support-oriented activities (Table 2). Though facilitators are trained in CTI, they do not provide CTI clinical support or case management training; clinical and training needs are referred to the REP team.

**Table 2:** Sample external facilitation activities

Implementation-oriented activities	Support-oriented activities
• Identify CTI implementation challenges and apply rapid-cycle improvement processes to address these challenges • Use VA administrative data to rapidly monitor outcomes (e.g., service use) of Veterans on a case manager’s caseload and provide feedback to aftercare staff • Educate aftercare staff and VA aftercare liaisons and identify key VA and community resources • Develop a site-specific CTI implementation plan	• Build relationships with aftercare staff • Encourage CTI practice and implementation • Educate aftercare staff on external facilitation, including its benefits and roles • Engage with and develop a plan to routinely update local change agents and other key stakeholders on CTI implementation • Ensure resources and personnel are available to grow and/or adapt CTI as needed

### Participating sites

Our partners at the GPD National Program Office identified 7 priority VA regions (Veterans Integrated Services Networks [VISNs]) for CTI implementation in aftercare. We will engage with VA homeless program leaders in these VISNs, and their associated VA facilities, to identify 32 aftercare sites across three cohorts, with 10–11 sites per cohort. Sites for each cohort will be identified in the 3 months prior to cohorts’ implementation initiation, based on sites self-identifying as being ready for implementation. Of note, our sample size (32 sites) was pre-determined by our funder.

### Study design

**Fig. 5:**
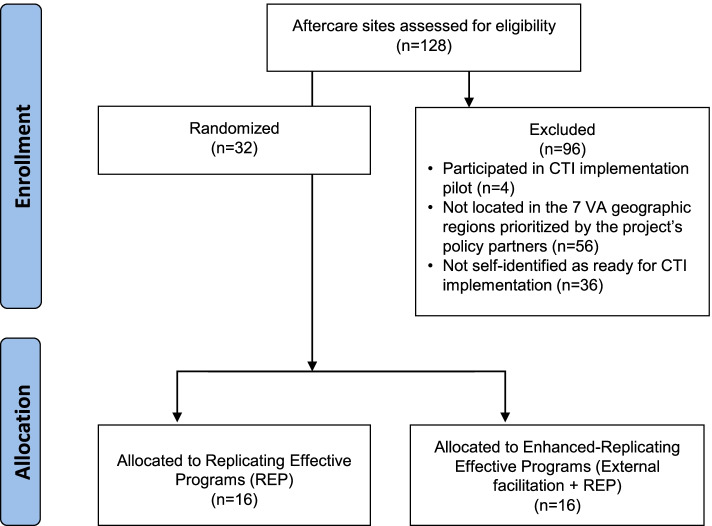
Consort diagram

We plan a type 3 hybrid implementation-effectiveness trial [13], registered as NCT05312229 on April 4, 2022, and determined to be non-research by VA’s Central Institutional Review Board. Given distinct leadership, staffing, and policies by site, and the high likelihood of contamination among case managers within sites, cluster randomization will occur at the site level. Within each cohort, the implementation team will use permuted block randomization—with geographic setting (urban, suburban, and rural) and number of case managers (a proxy for site size and resources) as stratification factors—to balance these factors among sites receiving REP versus enhanced REP. Figure 5 depicts a Consolidated Standards of Reporting Trials (CONSORT) flow diagram of our comparison groups. We will also use the stratification factors to match each of the 32 implementation sites to a control aftercare site not implementing CTI. Data from control sites will be used to assess selected quality metrics and costs of CTI as implemented. The CONSORT 2010 checklist of information for randomized controlled trials is available as supplemental material.

**Fig. 6:**
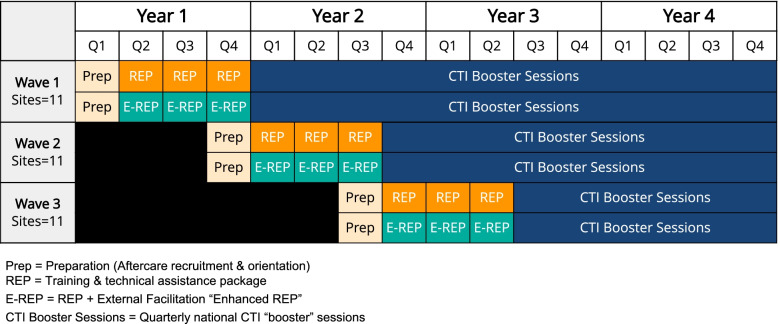
Staggered parallel clustered randomized trial design to assess implementation outcomes

We will use a staggered parallel cluster randomized design [23] (Fig. 6), with cohorts initiating REP and enhanced REP in 9-month intervals. Staggering is necessary to create cohorts sized for optimal training and EF within project resources. Sites are not blinded to implementation strategy assignments.

### Evaluation of CTI’s implementation and effectiveness

We plan a mixed-method evaluation of CTI’s implementation and effectiveness, to (a) capture fidelity to REP and enhanced REP; (b) compare CTI fidelity and sustainment achieved by REP versus enhanced REP; (c) compare quality metrics (housing stability, hospitalizations and other service use, and veteran and case manager experience) achieved by the two strategies and by CTI; (d) compare costs and return on investment (ROI) for CTI and the two implementation strategies; and (e) assess contextual factors that affect fidelity, sustainment, and quality metrics. Below, we describe the methods for each of these goals, followed by the methods for qualitative data collection and analyses that cut across goals.

### Implementation strategy fidelity

Fidelity to REP and enhanced REP will be assessed in three domains: [24] adherence (the extent to which strategies took place), dose (proportion of providers receiving each strategy), and responsiveness (provider receptivity to and involvement in strategies).

We will develop and maintain an Implementation Activity Log that summarizes all activities pertaining to REP and enhanced REP. The log will include dates and times of all training, TA, and EF sessions; participants; and templated notes on the implementation process. Adherence to REP and enhanced REP will be assessed by comparing the log to the processes described in the implementation strategies above. The dose will be captured by describing the frequency of TA/case consultation and EF sessions delivered to each site (and % of eligible participants attending). Responsiveness will be characterized by analyzing data from log notes, periodic reflections [25] (brief, ethnographically informed, guided discussions that capture near-real-time snapshots of implementation context), and semi-structured interviews with aftercare staff at 12 months post-implementation.

### CTI fidelity

We will measure site-level CTI fidelity 12 months after REP initiation, capturing adherence to CTI’s core components in the 6 months prior to measurement. We will employ methods adapted from a study of CTI for homeless adults leaving shelters [22] and developed with experts who have measured CTI fidelity across RCTs [22, 26]. Fidelity assessors will use a roster of HEVs engaged at implementation sites to randomly select 5 HEVs (“exemplars”) on each case manager’s caseload. Videoconferences with each case manager will be used to complete a templated review form by reviewing charts from these exemplars. Data from the exemplar review template will be used by the fidelity assessors to complete and score an adapted version of the CTI Implementation Self-Assessment Tool [27], which has cut-offs for adequate versus inadequate fidelity. We will use the *t*-test to compare fidelity between sites receiving REP versus enhanced REP.

These scores will be integrated with analyses of semi-structured aftercare staff interviews at 12 months and periodic reflections; qualitative data will glean staff perceptions of CTI fidelity. Within and across implementation strategies, we will compare sites with quantitative scores indicating adequate versus inadequate fidelity and use targeted coding of qualitative data to explore contextual factors associated with fidelity ratings.

### CTI sustainment

We will repeat our fidelity assessment 18 months after the start of implementation as a measure of CTI sustainment at 6 months after implementation supports cease. These scores will be integrated with analyses of semi-structured interviews with aftercare staff at 18 months and periodic reflections to explore perceptions of sustainment and contextual factors associated with sustainment across sites and implementation strategy assignment.

### Housing stability and hospitalizations

We will assess housing stability and hospitalizations among HEVs during and after receiving aftercare services, comparing: (a) REP versus enhanced REP sites and (b) the 32 implementation sites with a control group of 32 sites that did not implement CTI, matched on geographic setting and number of case managers. We will use administrative data (from VA’s Corporate Data Warehouse [CDW] and homeless registry) to assess these outcomes (Table 3).

**Table 3:** Summary of quality metrics assessed using VA administrative data

Domain	Quality metric
Housing stability	• Number of days between aftercare entry and first VA indicator of housing instability^a^ • Annual number of encounters with VA homeless services • Annual number of discrete episodes^b^ of engagement with VA homeless services
Hospitalizations	• Number of days between aftercare entry and first hospitalization (VA or non-VA) • Annual number of hospitalizations (total and separated into medical/surgical versus mental health) • Annual number of hospital bed days
Outpatient service use	• Primary care provider assignment (yes/no) • Presence or absence of at least one primary care visits/year • Use of VA vocational services (among HEVs seeking employment) • Total number of mental health visits (among HEVs with mental health disorders) • Total number of substance use disorder treatment visits (among HEVs with substance use disorders)

^a^Use of VA homeless services

^b^New episodes desfined by 30 days passing between encounters

For all HEVs who enter implementation and control sites between 6 and 18 months post-implementation (the time during which we have captured fidelity), we will determine the number of days between aftercare entry and the first VA indicator of housing instability (i.e., homeless service use). Within each cohort, for sites that receive REP or enhanced REP to support CTI implementation, we will use survival analysis (Cox proportional hazards models) to compare time to the first presence of a housing instability indicator among those who received aftercare in the same period in matched control sites. Models will use clustered robust standard errors to account for potential within-site correlation. We estimate that there will be 4,080 HEVs split evenly between implementation and control sites (~64 HEVs/site). Using a two-sided alpha level of 0.05, we estimate having 80% power to detect a 25% relative difference in time to housing instability indicators. This estimate is based on a log-rank test, assuming an intraclass correlation within each site of ~0.05.

Within implementation sites, we will assess for independent associations between housing instability among HEVs and sites’ assigned implementation strategy (REP versus enhanced REP) and CTI fidelity. We will also compare HEVs between implementation and control sites; per HEV, we will compare the annual number of encounters with VA homeless services (a proxy for service intensity needed) and the annual number of episodes of homeless service engagement (with new episodes defined by ≥30 days between encounters). Comparisons will be made using negative binomial regression models with clustered robust standard errors for site-level clustering.

Similarly, we will determine, for each HEV, the days between entry into aftercare and hospitalization (at the VA or paid for by VA at an outside hospital) and describe the time to hospitalization using Kaplan-Meier curves. We will include all hospitalization types (mental health, medical/surgical), analyzing the time to any hospitalization and by type. We will also assess for independent associations between these outcomes and implementation strategy and CTI fidelity using Cox proportional hazards models. Additional secondary outcomes will be the annual number of hospitalizations (total and by type) and the number of bed days per HEV. We will also assess time trends in housing stability and hospitalizations within implementation sites, comparing pre- and post-implementation.

### Outpatient service use

As decreased hospitalizations are often linked with increased outpatient service use, we expect that HEVs who receive CTI will have increased engagement with outpatient VA mental health, SUD, primary care, and vocational services and decreased emergency department (ED) use. We will use VA administrative data to compare the use of these services (Table 3) among HEVs in aftercare with diagnoses indicating that they would benefit from such care at sites receiving REP versus enhanced REP, as well as implementation versus control sites.

Using these same comparison groups, we will assess differences in primary care engagement using established VA benchmarks (i.e., primary care provider assignment and having ≥1 visit/year). For all HEVs noted in the aftercare roster to be seeking employment, we will assess the use of VA vocational services. For HEVs with mental health disorders or SUDs noted by the International Classification of Diseases (ICD)-10 codes associated with inpatient and outpatient VA care, we will assess and compare their engagement with outpatient mental health and/or SUD services, respectively (i.e., the total number of visits, number of quarters with ≥1 visit).

### HEV and case manager experiences

We will conduct semi-structured interviews with HEVs at implementation sites at baseline and 18 months, characterizing and comparing HEVs’ experiences of and satisfaction with aftercare pre- and post-CTI implementation. We will use semi-structured interviews with aftercare staff at implementation sites (at baseline and 12 months) to characterize case manager experiences with REP and/or enhanced REP and experiences of and satisfaction with providing aftercare services pre- and post-CTI implementation. We will assess for differences in staff experiences between sites that received REP versus enhanced REP. Data from these interviews will be integrated with periodic reflections to assess contextual factors and implementation strategy components associated with the valence of experiences described by HEVs and case managers.

### Costs and return-on-investment (ROI)

We will estimate the costs and ROI of CTI implementation in aftercare from the perspective of VA; costs associated with non-VA homeless and healthcare services are outside the scope of these analyses. We will compare costs at sites that receive REP versus enhanced REP and calculate the ROI of CTI implementation, as compared to control sites. In addition to implementation costs, CTI may lead to increased VA costs associated with enhanced outpatient service use but decreased costs associated with inpatient care. There may also be decreased costs from VA homeless services as veterans retain permanent housing.

#### Assessing CTI costs as actually implemented

The predominant cost of delivering CTI in aftercare is case manager time. Case managers are funded by the GPD National Program Office; we anticipate that case managers’ activities will be reorganized to deliver CTI, increasing their efficiency without additional costs. To test this, we will compare the mean monthly cost of CTI case management per aftercare HEV served to the mean monthly cost at aftercare control sites.

We will obtain estimates of case manager salary and benefits from the GPD National Program Office. Then, using a roster of HEVs at each aftercare site, we will determine the average number of HEVs served per case manager per month between 6 and 18 months post-CTI implementation. For each case manager, we will calculate the cost per HEV served per month, comparing implementation versus control sites. In addition, data from 12-month aftercare staff interviews will be used to glean contextual factors that may influence caseload and case management activities.

#### Costs of implementation strategies

We will compare the costs of using REP versus enhanced REP for spreading and sustaining CTI. We conceptualize the adaptation of CTI to aftercare as a fixed, non-repeatable cost that will not be included in our analyses. Most costs associated with the spread stage of implementation are fixed, repeated with each cohort, including CTI initial training, communities of practice, and toolkit maintenance; these costs will be assessed by cohort. Variable spread costs that depend on the number of sites per cohort include case consultation and EF; these will be assessed by the site. With respect to sustainment, the quarterly booster sessions and re-training of new case managers at the participating implementation sites are repeatable fixed costs. Case manager turnover will be tracked using the national roster of aftercare participants per case manager.

All implementation activities of our team will be tracked through the Implementation Activity Log. In addition, to capture time spent by the implementation team on session preparation or documentation (i.e., time that is not captured in the Implementation Activity Log), team members will complete cost capture templates weekly during the second implementation cohort. Implementation activity log and cost capture template data will be linked to hourly costs per team member.

#### ROI of CTI and implementation strategies

The financial ROI of CTI and these implementation strategies depends on savings reaped through improved housing stability and decreased hospitalizations. To calculate the degree of cost offsets, we will link HEVs’ use of VA housing and health services to the costs of these activities which are available in an activity-based cost allocation system (the Managerial Cost Accounting System) [28]. Among HEVs who enroll at implementation sites between 6 and 18 months after the initiation of CTI implementation, we will calculate mean housing and health services costs per HEV in the 6, 12, and 18 months after enrollment. We will use a multivariable generalized linear model and two-part regression models where appropriate to compute ROI at 6-, 12-, and 18-month time horizons for HEVs in sites that received REP versus enhanced REP, and compared to the 32 control sites. We will compare model fit using a generalized linear model and ordinary least squares [29].

### Contextual factors

Using data from semi-structured interviews with aftercare case managers and supervisors, and periodic reflections with aftercare liaisons and Housing Transitions QUERI staff engaged in implementation, we will assess CFIR-based contextual factors relevant to CTI, including provider and site characteristics, inner setting and outer context, perceived veteran needs, and perceived characteristics of CTI, REP, and enhanced REP. We will integrate these data with our assessments of fidelity, sustainment, quality metrics, costs, and ROI to explore potential relationships.

### Qualitative data collection

Table 4 summarizes our planned qualitative data collection, detailed below.

**Table 4:** Summary of qualitative data collection

	Sample size	Timing^**a**^	Duration
**Interviews**			
Aftercare case managers and supervisors	50 (16–17/cohort)	Baseline^b^, 12 and 18 months	45 min
HEVs enrolled pre-CTI implementation	30 (10/cohort)	Baseline	30–45 min
HEVs enrolled post-CTI implementation	30 (10/cohort)	18 months	
**Periodic reflections**			
Implementation team	~6	Monthly	15–30 min
Aftercare liaisons	18 (6/cohort)	Quarterly	

^a^In relationship to each cohort’s CTI implementation initiation

^b^Post-initial CTI training but prior to implementation

#### Aftercare staff semi-structured interviews

As described above, we will conduct semi-structured interviews with aftercare staff (~50, or 16–17/cohort, including liaisons, supervisors, and case managers) engaged in CTI implementation across the 32 sites. We will use maximum variation sampling [30] to ensure diversity by geography (urban, suburban, rural) and the number of case managers per site. Interviews will be conducted, by cohort, at baseline (during and post-CTI training) and 12 and 18 months post-implementation. At baseline, we will assess staff background, education, and training; baseline practices; contextual factors (e.g., site-specific characteristics); and knowledge, prior experiences with and perceptions of CTI, and expectations about CTI implementation barriers and facilitators. Interviews at 12 and 18 months will assess experiences with CTI and with REP and/or enhanced REP, fidelity to CTI’s core components, and recommendations for CTI implementation support. Interviews at 18 months will additionally assess barriers and facilitators to sustainment. Each interview will last ~45 min and will be conducted by videoconference or telephone.

#### HEV semi-structured interviews

We will use the GPD National Program Office’s aftercare roster to identify up to 30 HEVs (10/cohort) who used aftercare in the 6 months prior to implementation, and a different set of 30 HEVs (10/cohort) who used aftercare 12–18 months after implementation. We will use maximum variation sampling [30] to ensure diversity by site and age. We will assess perceived needs from and experiences with the aftercare program. These ~30–45-min interviews will be conducted by telephone.

#### Periodic reflections

Periodic reflections are ethnographically informed, pragmatic, and a low-burden method to observe implementation events and contextual factors among stakeholders over the implementation period. These reflections support consistent documentation of implementation strategies, adaptations, changes to inner and outer settings, unexpected events, and experiences of EBP implementation [25]. Periodic reflections will be conducted monthly with Housing Transitions QUERI staff engaged in CTI implementation and quarterly with a subset (*n*=18, 6 per cohort) of VA liaisons to the aftercare program. We will purposively sample liaisons affiliated with urban, suburban, and rural sites. All reflections (15–30 min/each) will be conducted by telephone or videoconference.

#### Analyses

Analyses of interview and reflection data will employ rapid qualitative analyses [31]. We will create a structured summary for each interview or reflection using templates organized by domains of interest within our conceptual framework; we will develop matrices to synthesize content by site, cohort, and participant, before completing summary tabulation tables to validate patterns identified in the data and ensure the trustworthiness of findings. We will conduct targeted in-depth coding and analyses as needed, using the constant comparison method [32] and ATLAS.ti software to confirm/disconfirm exploratory hypotheses or explore emergent data patterns. Qualitative and quantitative data will be gathered concurrently over the course of implementation and integrated to identify whether/how contextual factors emerging in qualitative data are associated with quantitative implementation outcomes.

### Products to support the continued spread and sustainment of CTI in aftercare

Though the scope of this project is to implement CTI in 32 sites, our overall goal is to support our partners in using knowledge from this project to implement and sustain CTI in all 128 aftercare sites. Therefore, we will package the findings and products from this project into a business case analysis and implementation playbook.

### Business case analysis

We will use findings from our implementation evaluation to prepare a business case analysis of the costs, cost offsets, and non-financial benefits of CTI sustainment and spread in aftercare and the business case for REP versus enhanced REP. These analyses will be prepared from the perspective of VA’s Homeless Program Office, which oversees aftercare and pays for its services. Table 5 displays expected CTI costs (for spread/sustainment and care delivery) and benefits (cost offsets and non-financial evaluation). Our evaluation estimates the magnitude and features of these costs and benefits. We will assess whether enhanced REP’s investment in EF yields a sufficient combination of downstream financial cost offsets and non-financial benefits to justify enhanced REP as the preferred strategy, over REP alone, to spread and sustain CTI. We will also provide information about aftercare contextual factors that may signal an above-average benefit from enhanced REP versus REP.

**Table 5:** Expected resources and benefits to be measured for CTI spread and sustainment

Expected resources (costs)		Expected benefits
Spread/Sustainment costs	Care delivery costs	Cost offsets and non-financial benefits
• *Fixed*: Implementation team providing 6-week CTI training, aftercare staff and VA aftercare liaison receiving a 6-week CTI training, Community of Practice webinars, toolkit maintenance, and quarterly CTI booster sessions • *Variable*: Implementation team and aftercare staff time spent giving/receiving case consultation and EF^a^	• *Variable:* Aftercare staff time spent providing CTI^b^; VA provider time associated with increased outpatient mental health, SUD, medical, and vocational service use	• *Cost offset:* decreased use of VA residential housing services; decreased hospitalizations • *Non-financial benefits*: HEVs’ housing stability; HEVs’ receipt of needed mental health, SUD, medical and vocational services; improved HEVs’ care experiences; improved case managers’ experiences providing care

^a^Only applies to aftercare sites receiving enhanced REP to support CTI implementation and sustainment

^b^If there are additional costs associated with using CTI and/or the policy partners were to stop paying for this staff

### Implementation playbook

We will also develop an online implementation playbook [33, 34], a user-friendly compendium of CTI implementation processes, targets, and outcomes. The playbook will be developed iteratively and collaboratively with our partners to ensure an optimal match between content and partner needs. It will serve as a “how to” guide for spread and sustainment.

The playbook centerpiece will be the finalized CTI package with products generated across REP (e.g., training manuals) and enhanced REP (e.g., facilitation guide). We will iteratively develop guides for these products. The qualitative data collected will provide insights into the nuances of using playbook components and highlight potential adaptations to meet sites’ contextual factors. We will also include implementation “tip sheets,” i.e., small, targeted, informational bytes, to facilitate CTI spread and maintenance. The playbook will also contain copies of leadership briefings and frequently asked questions.

## Discussion

To accelerate VA’s efforts to end Veteran homelessness, we must enhance the implementation and sustainment of EBPs among VA and community providers who serve HEVs. In aftercare, national CTI implementation holds the potential to improve housing and health for one of VA’s most vulnerable populations. Moreover, as case management is one of the primary services offered to HEVs, this project aims to advance the science surrounding strategies that support the implementation and sustainment of effective case management practices. For CTI to be successful at a given aftercare site, an appropriate implementation strategy must be used, considering contextual factors that support and/or impede implementation.

We will use REP to ensure that a robust and stakeholder-informed CTI training and TA package is delivered across 32 aftercare sites. In some settings and contexts, this package may be sufficient to enable CTI implementation and evaluation. However, our cluster-randomized design will allow us to evaluate the business case for adding EF and suggest site-level features that may benefit from this strategy augmentation. Our rollout design, staggering sites’ implementation start dates, allows for a comparative effectiveness-implementation trial of active base and enhanced implementation strategies, balancing training needs with project resources [23]. Though we face implementation challenges—including high rates of staff turnover within human service organizations [35]—we selected a dynamic base implementation strategy that is built on stakeholder engagement [12], which is important in implementation approaches to address homelessness [10].

We note that this implementation initiative was developed in close collaboration with our policy partners; this protocol describes an initiative within a specific VA homeless program, operated by diverse community-based organizations. As such, the findings of our planned implementation-effectiveness evaluation may not extrapolate to other VA or non-VA settings; rather, our goal is to produce products that allow for the continued spread in this setting. The selected sample size (*n*=32) was predetermined by our funder as opposed to deriving from power analyses. Moreover, our selected implementation strategies are robust and stakeholder-informed training and technical assistance (REP and enhanced REP), and external facilitation (enhanced REP); these are costly and time-intensive strategies to support EBP implementation and may not be feasible in low-resource settings.

Regardless of these limitations, for HEVs in aftercare, CTI holds promise as an EBP that can improve this population’s housing and health. The products planned for our policy partners (i.e., business case analysis and implementation playbook) will provide a blueprint for spread to the remainder of aftercare sites, acknowledging the important role of contextual factors in implementation strategy selection. Moreover, understanding effective strategies to support CTI implementation could also support the implementation of other case management practices within and outside VA.

## Data Availability

Not applicable

## Abbreviations

CDW: Corporate Data Warehouse
CFIR: Consolidated Framework for Implementation Research
CONSORT: Consolidated Standards of Reporting Trials
COVID-19: Coronavirus disease 2019
CTI: Critical time intervention
EBP: Evidence-based practice
ED: Emergency department
EF: External facilitation
GPD: Grant and Per Diem
HEV: Homeless-experienced veterans
ICD: International Classification of Diseases
PII: Partnered Implementation Initiative
QUERI: Quality Enhancement Research Initiative
REP: Replicating Effective Programs
ROI: Return on investment
SUD: Substance use disorder
TA: Technical assistance
U.S.: United States
VA: Veterans Health Administration
VISN: Veterans Integrated Services Network
